# A short scale to measure health-related quality of life after traumatic brain injury in children and adolescents (QOLIBRI-OS-KID/ADO): psychometric properties and German reference values

**DOI:** 10.1007/s11136-024-03764-3

**Published:** 2024-08-31

**Authors:** Marina Zeldovich, Leonie Krol, Inga K. Koerte, Katrin Cunitz, Matthias Kieslich, Marlene Henrich, Knut Brockmann, Anna Buchheim, Michael Lendt, Christian Auer, Axel Neu, Joenna Driemeyer, Ulrike Wartemann, Claudius Thomé, Daniel Pinggera, Steffen Berweck, Michaela V. Bonfert, Joachim Suss, Holger Muehlan, Nicole von Steinbuechel

**Affiliations:** 1 Institute of Psychology, Faculty of Psychology and Sport Science, University of Innsbruck, Innsbruck, Austria; 2 Faculty of Psychotherapy Science, Sigmund Freud University, Vienna, Austria; 3 Department of Psychology, Clinical Psychology and Psychotherapy, Philipps University of Marburg, Marburg, Germany; 4 cBRAIN / Department of Child and Adolescent Psychiatry, Psychosomatics, and Psychotherapy, LMU University Hospital, Ludwig-Maximilian University, Munich, Germany; 5 Psychiatry Neuroimaging Laboratory, Department of Psychiatry, Mass General Brigham, Boston, USA; 6 Department of Psychiatry and Psychotherapy, University Medical Center Goettingen, Goettingen, Germany; 7 Department of Paediatric Neurology, Hospital of Goethe University, Frankfurt, Germany; 8 Interdisciplinary Pediatric Center for Children with Developmental Disabilities and Severe Chronic Disorders, Department of Pediatrics and Adolescent Medicine, University Medical Center, Goettingen, Germany; 9 Neuropediatrics, St. Mauritius Therapeutic Clinic, Meerbusch, Germany; 10 Department of Neurosurgery, Kepler University Hospital GmbH, Johannes Kepler University Linz, Linz, Austria; 11 Clinical Research Institute für Neurosciences, Faculty of Medicine, Johannes Kepler University Linz, Linz, Austria; 12 Department of Neurology and Neuropediatry, VAMED Klinik Geesthacht GmbH, Geesthacht, Germany; 13 Department of Pediatrics, University of Hamburg-Eppendorf, Hamburg, Germany; 14 Department of Neuropediatrics, VAMED Klinik Hohenstücken GmbH, Brandenburg an der Havel, Germany; 15 Department of Neurosurgery, Medical University lnnsbruck, Innsbruck, Austria; 16 Specialist Center for Paediatric Neurology, Neurorehabilitation and Epileptology, Schoen Klinik, Vogtareuth, Germany; 17 Department of Pediatric Neurology and Developmental Medicine and LMU Center for Development and Children with Medical Complexity, Dr. Von Hauner Children’s Hospital, LMU University Hospital, Munich, Germany; 18 Department of Pediatric Surgery, Wilhelmstift Catholic Children’s Hospital, Hamburg, Germany; 19 Department of Health and Prevention, University of Greifswald, Greifswald, Germany

**Keywords:** Pediatric traumatic brain injury (pTBI), Health-related quality of life (HRQoL), Reference values, Patient-reported outcome measure (PROM)

## Abstract

**Purpose:**

The impact of pediatric traumatic brain injury (pTBI) on health-related quality of life (HRQoL) in children and adolescents remains understudied. Short scales have some advantages in terms of economy and administration over longer scales, especially in younger children. The aim of the present study is to psychometrically evaluate the six-item German version of the QOLIBRI-OS-KID/ADO scale for children and adolescents. In addition, reference values from a general German pediatric population are obtained to assist clinicians and researchers in the interpretation of HRQoL after pTBI.

**Methods:**

A total of 297 individuals after TBI and 1997 from a general population sample completed the questionnaire. Reliability, validity, and comparability of the assessed construct were examined.

**Results:**

The questionnaire showed satisfactory reliability (α = 0.75 and ω = 0.81 and α = 0.85 and ω = 0.86 for the TBI and general population samples, respectively). The QOLIBRI-OS-KID/ADO was highly correlated with its long version (R^2^ = 67%) and showed an overlap with generic HRQoL (R^2^ = 55%) in the TBI sample. The one-dimensional factorial structure could be replicated and tested for measurement invariance between samples, indicating a comparable HRQoL construct assessment. Therefore, reference values and cut-offs indicating clinically relevant impairment could be provided using percentiles stratified by factors significantly associated with the total score in the regression analyses (i.e., age group and gender).

**Conclusion:**

In combination with the cut-offs, the QOLIBRI-OS-KID/ADO provides a cost-effective screening tool, complemented by interpretation guidelines, which may help to draw clinical conclusions and indications such as further administration of a longer version of the instrument to gain more detailed insight into impaired HRQoL domains or omission of further steps in the absence of an indication.

**Supplementary Information:**

The online version contains supplementary material available at 10.1007/s11136-024-03764-3.

## Introduction

Pediatric traumatic brain injury (pTBI) is a global health problem affecting millions of children and adolescents worldwide [1]. It can have a profound impact on multiple aspects of a young individual’s life, including their physical, cognitive, emotional, and social well-being [2]. These outcomes are directly related to health-related quality of life (HRQoL), which remains understudied in pTBI patients [3].

To date, only generic HRQoL measures have been available for use in the pTBI context. However, such generic measures, while useful for comparisons between different disease populations [4], have been found to be too general to sensitively capture the outcome of a specific health condition [5]. Disease-specific HRQoL instruments, on the other hand, provide a more comprehensive assessment by capturing the individual aspects of life affected by a particular disease [6]. Until now, HRQoL after pTBI has been assessed using either generic instruments such as the Pediatric Quality of Life Inventory [7] as a self-report measure, or, more recently, the disease-specific Traumatic Brain Injury Quality of Life Communication Item Bank [8] using the parent perspective, which covers however functional communication only. Given the advantages of disease-specific measures over generic ones, the ability of children to evaluate their own HRQoL [9], and the fact that parent–child assessments of HRQoL are not always congruent [10–12], age-appropriate TBI-specific patient-reported outcome measures (PROM) are needed.

To fill this gap, the 35-item self-report Quality of Life after Brain Injury for children and adolescents (QOLIBRI-KID/ADO) questionnaire was developed as the first instrument to assess HRQoL in German-speaking children and adolescents after pTBI [13, 14]. It corresponds to the 37-item adult version (but omits items on satisfaction with sexual life and finances) [15], covers six domains (Cognition, Self, Daily Life & Autonomy, Social Relationships, Emotions, and Physical Problems) that are particularly relevant for monitoring after (p)TBI [15], and aims to assess TBI-specific HRQoL in the pediatric context and to measure the burden of pTBI across the lifespan.

One of the notable advantages of short scales is their economy and ease of administration. These features are particularly important when working with pediatric populations, as their attention span and fatigue tolerance may be limited in general and after pTBI in particular [2]. The development and use of a short overall scale (i.e., QOLIBRI-OS-KID/ADO) may therefore facilitate the collection of information and allow for administration after more severe pTBI without placing additional burden on the young patients. The six-item adult version has already demonstrated robust psychometric properties [16] and high sensitivity to different patient groups [17], making it an attractive tool in both clinical and research settings. The applicability of the overall scale in adults underscores the potential of a similar measure for children and adolescents. By utilizing a short and valid instrument such as the six-item QOLIBRI-OS-KID/ADO questionnaire, clinicians can efficiently assess the comprehensive aspects of HRQoL dimensions in pTBI patients in a condensed form (i.e., by rating overall satisfaction within each domain), thereby optimizing resources and minimizing patient burden.

The availability of reference values from comparable general populations facilitates the interpretation of questionnaire scores obtained from individual patients. A comparison of the level of HRQoL after pTBI with the level of HRQoL in the general pediatric population will provide a more realistic assessment of the post-injury burden.

With these considerations in mind, this study aims to investigate the psychometric properties and the applicability of the recently developed German six-item QOLIBRI-OS-KID/ADO questionnaire. The objectives are to examine the psychometric properties within the framework of classical test theory and to establish reference values based on a German general population sample of children and adolescents.

## Methods

### Participants

#### pTBI sample

**Fig. 1:**
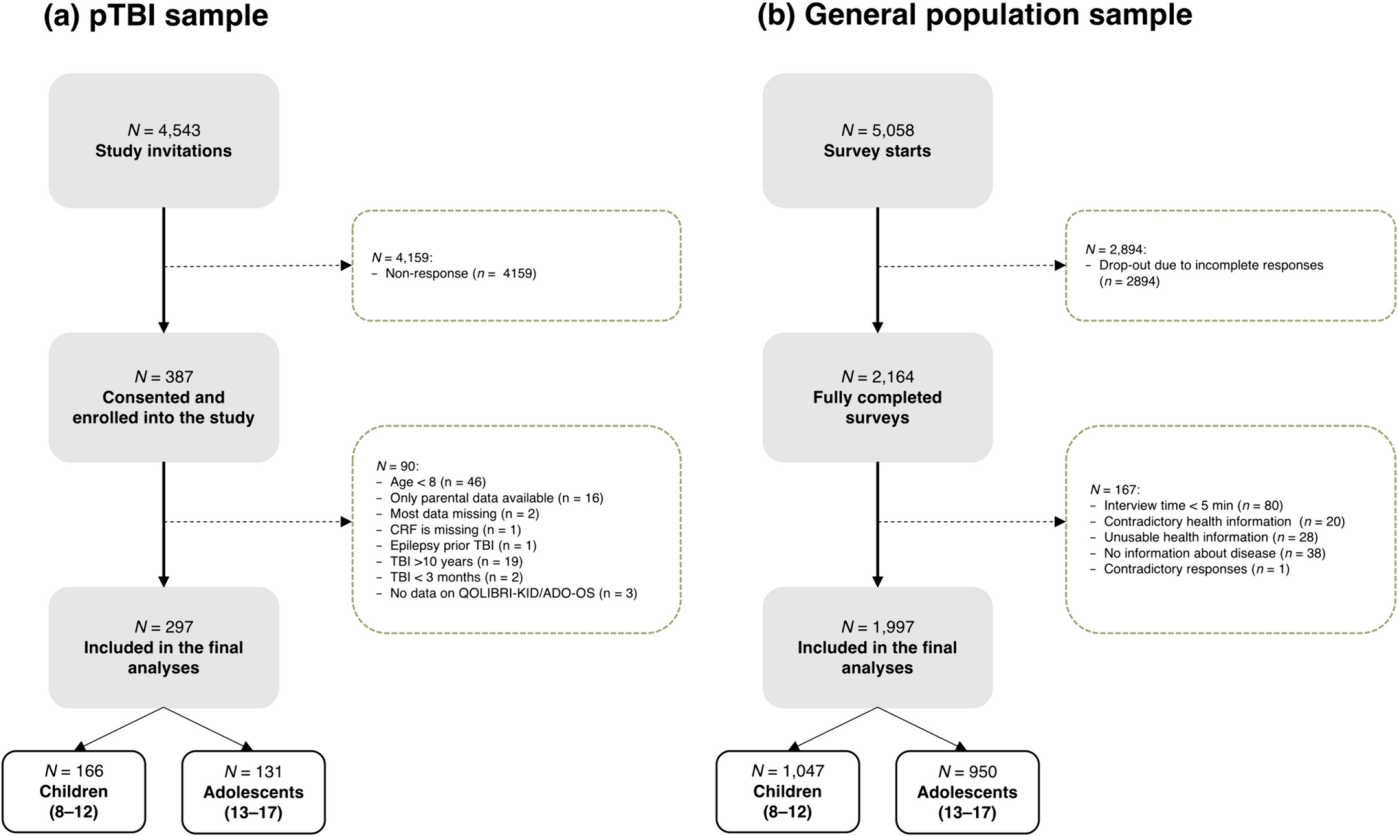
Sample attrition for the **a** pTBI sample and for the **b** general population sample

Individuals after pTBI were recruited based on administrative databases of 12 medical centers in Germany and Austria between February 2021 and February 2022. Medical records of potential participating children and adolescents were retrieved from the databases and screened for eligibility. Participants were eligible to participate if they were 8–17 years of age, had been diagnosed with pTBI three months to ten years prior to enrollment, had information on pTBI severity (either based on Glasgow Coma Scale score [18] or medical reports), and were able to understand and respond to questions in German. Children and adolescents with pre-injury epilepsy, very severe polytrauma, severe premorbid mental illness, or life-threatening health conditions were not eligible to participate. Mail invitations were sent to the parents or legal guardians of children and adolescents who met the inclusion criteria. These invitations explained the purpose of the study, the setting, and contact information for study participation. Written informed consent was obtained from all participants and/or legal guardians at the time of study enrollment if parents consented to participate. Interviews with children and adolescents were conducted face-to-face, with the assistance of trained investigators, at separately arranged appointments (online or on-site). Parents completed paper–pencil versions of the questionnaires. A priori power analyses indicated that the primary objective of the project (i.e., to develop the long version of the TBI-specific HRQoL instrument for children and adolescents and to test its psychometric properties) would require a sample of approximately 140 individuals per age group [19]. A total of 297 children and adolescents were included in the analyses (Fig. 1a).

#### General population sample

Individuals from general population were recruited via an online survey between March 2022 and April 2022 with the assistance of two international market research agencies based in Germany (Dynata: https://www.dynata.com; respondi: https://www.respondi.com; last accessed 05.07.2024). The agencies used their databases to identify and contact parents of children and adolescents aged 8–17 years. Parents were first informed of the purpose of the data assessment and the privacy policy and were asked to provide consent to assessing of sensitive information (i.e., their children’s health data). They were asked if their child had experienced pTBI or any other life-threatening condition. If they responded affirmatively, study participation was terminated. Otherwise, they continued to answer sociodemographic questions and were then asked about their child’s availability. If the child was available, they were invited to participate, or the survey could be continued later. As an incentive, participants received tokens or certificates. The planned sample size was N = 2000 with an even distribution of children and adolescents to obtain robust reference values, taking into account that stratification by other characteristics such as gender may be necessary.

To ensure the data quality, participants who provided inconsistent or unusable information (e.g., conflicting statements of being both completely satisfied and completely bothered), those with contradictory responses (e.g., reporting no health problems but providing comments in the text box), and those who completed the survey in less than five minutes were excluded. A total of 1997 children and adolescents were included in the analyses (Fig. 1b).

### Instruments

The *Quality of life after brain injury for children and adolescents – overall scale* (QOLIBRI-OS-KID/ADO) is a six-item PROM that assesses the *overall* satisfaction and corresponds to the adult version [16]. The items relate to five out of the six domains (i.e., Physical problems, Cognition, Emotions, Autonomy, Social aspects) of the 35-item version, the QOLIBRI-KID/ADO [13, 14], supplemented by a question on Future prospects. Answers are to be rated on a five-point Likert-type scale (from “Not at all” to “Very”) with a two-week recall period. The scale is accompanied by smileys, which make it easier to rate, especially for younger children. The total score is calculated as the mean of the item responses, transformed into a scale ranging from 0 (lowest possible HRQoL) to 100 (highest possible HRQoL). The latter is done by subtracting 1 from each item score and multiplying by 25. Supplementary material provides information on the development of the questionnaire and the final wording (Development of the QOLIBRI-OS-KID/ADO and Table S1). For administration to the general population, the reference to injury was removed from the instructions. Table 1 summarizes information on further instruments administered in the study.

**Table 1:** Overview of further instruments administered in the study

Instrument	Outcome domain	Type	Response scale	Scales (# items)	Scores	Cut-offs applied
Quality of life after brain injury for children and adolescents (QOLIBRI-KID/ADO) [13]	TBI-specific HRQoL	Self-report	Five-point Likert-type (“Not at all” to “Very”)	Cognition (7) Self (5) Daily Life & Autonomy (7) Social Relationships (6) Emotions (4) Physical Problems (6) Total score (35)	0 (lowest possible HRQoL) to 100 (highest possible HRQoL)	None
Pediatric quality of life inventory (PedsQL™) [7]	Generic HRQoL	Self-report	Four-point Likert-type scale (“Never” to “Almost always”)	Emotional Functioning* (5) Social Functioning* (5) School Functioning* (5) Physical Functioning (8) Psychosocial Health Summary Score (sum of 15 items from the scales marked with *) Total score (23)	0 (lowest possible HRQoL) to 100 (highest possible HRQoL)	None
Generalized anxiety disorder 7 (GAD-7) [20]	Generalized anxiety disorder	Proxy-report	Four-point Likert-type scale (“Not at all” to “Nearly every day”)	Total score (7)	0 (no anxiety) to 21 (severe anxiety)	0–4 (no/minimal anxiety) vs. 5–21 (mild to severe anxiety) [21]
Patient health questionnaire 9 (PHQ-9) [22]	Major depression	Proxy-report	Four-point Likert-type scale (“Not at all” to “Nearly every day”)	Total score (9)	0 (no depression) to 27 (severe depression)	0–4 (no/minimal depression) vs. 5–27 (mild to severe depression) [23]
Postconcussion symptoms inventory—self-report (PCSI-SR8) [24]	Post-concussion symptoms	Self-report	Three-point Guttman response scale (“No problem” to “A lot of problem”)	Physical (8) Emotional (3) Cognitive (4) Sleep/fatigue (2) Total score (17)	0 (no symptoms) to 34 (maximum symptom intensity)	Below, above or within the mean range (M ± 1 SD)^1^
Postconcussion symptoms inventory—self-report (PCSI-SR13) [24]	Post-concussion symptoms	Self-report	Seven-point Guttman scale with three anchors (“'No problem”, “Moderate problem”, “Severe problem”)	Physical (8) Emotional (4) Cognitive (6) Sleep/fatigue (3) Total score (21)	0 (no symptoms) to 126 (maximum symptom intensity)	Below, above or within the mean range (M ± 1 SD)^a^
Rey auditory verbal learning test (RAVLT) [25, 26]	Ability to learn and remember verbal information	Performance-based	–	- (15 Words)	0 (no words recalled) to 15 (all words recalled) for each trial	Difference between number of words recalled in trial I and the number of words recalled in trial V classified as above, below, or within range based on the mean range (M ± 1SD) of the corresponding age group (i.e., 8–12 years and 13–17 years)^1^

*QOLIBRI-KID/ADO* quality of life after injury—children and adolescents, *PedsQL* pediatric quality of life inventory, *GAD-7* generalized anxiety disorder 7, *PHQ-9* patient health questionnaire 9, *PCSI-SR8/SR13* postconcussion symptoms inventory (*SR8* children aged 8–12 years, *SR13* adolescents aged 13–18 years), *RAVLT* Rey auditory learning test, *TBI* traumatic brain injury, *HRQoL* health-related quality of life

^a^In the absence of standard cut-off values or recent normative data, scores are categorized as above, below, or within range based on the mean range (M ± 1SD) per age group, as appropriate

### Sociodemographic and health-related information

In both samples, sociodemographic information included age, gender, and school attendance. In the pTBI sample, health-related information included parent-reported health status (presence of chronic health conditions, neurological disorders, and/or developmental health conditions), TBI severity (mild, moderate, severe), time since injury in years, and functional recovery status assessed by the King’s Outcome Scale for Childhood Head Injury (KOSCHI) [27] with the following categories: (3a/b) lower/upper severe disability, (4a/b) lower/upper moderate disability, and (5a/b) good/intact recovery.

In the general population sample, health status information was reported by parents on nine health domains: central nervous system disease, alcohol and/or psychotropic drug abuse, active or uncontrolled systemic disease, psychiatric disturbances, severe sensory disturbances, use of psychotropic or other drugs, intellectual disability or other neurobehavioral disturbances, problems before, during, and after childbirth, and other. A participant was considered to have at least one chronic condition if at least one domain was endorsed.

### Statistical analyses

To ensure comparability of the results of the QOLIBRI-OS-KID/ADO with its long version, we largely followed the analysis scheme of the QOLIBRI-KID/ADO study [13]. For descriptive analyses at the item level, we calculated mean (M), standard deviation (SD), skewness (SK), and frequency of item responses. Skewness was classified as absent (− 0.5 and 0.5), moderate (± 0.5 and ± 1), and high (> ± 1) [28]. For the QOLIBRI-OS-KID/ADO total score, we reported the M, SD, median (Mdn) and quartiles (Q1 and Q3).

Differential item functioning (DIF) analyses using a combination of ordinal logistic regression and item response theory (LORDIF) were conducted to investigate whether the aggregation of data from children (aged 8–12 years) and adolescents (aged 13–17 years) is appropriate. For each item, two LORDIF models were compared separately in the pTBI and the general population samples: Model 1 including ability (i.e., the level of the latent trait, HRQoL, measured by an item), and Model 2 including ability, age category, and the interaction between age category and ability. DIF was considered absent if a non-significant difference (p > 0.01) was found and the effect measured by McFadden’s pseudo R^2^ was < 0.05 [29]. In the absence of DIF, psychometric analyses can be conducted using aggregated data from children and adolescents.

The reliability was examined in both samples using Cronbach’s α and McDonald’s ω (for both characteristics, values ≥ 0.70 were considered desirable [30]). The reporting of McDonald’s ω, which is calculated based on the factorial structure of a measure and thus accounts for variation in the correlation strength between items and the latent factor [31], was chosen to further support the internal consistency results. We calculated Cronbach’s α while omitting each of the six items to ensure that the scale reliability did not increase when one of the items was removed. Associations between the items and the total score were explored using corrected item-total correlations (CITC > 0.40) [32]. Test–retest reliability was examined in a subsample of patients who completed the questionnaire on two occasions, 10 to 20 days apart. For this purpose, the intraclass correlation coefficient using a two-way random effects model (i.e., ICC(2,1)) was calculated on the total score level, with ICC > 0.60 [33] considered desirable. The standard error of measurement (SEm) [34] and the minimal detectable change (MDC) were calculated based on the ICCs to provide information on the score variability and the “true” change in the QOLIBRI-OS-KID/ADO total score.

Factorial validity was examined using confirmatory factor analysis (CFA) with the weighted least square (WLS) estimator in both the pTBI and the general population samples. The one-factor solution was considered to describe the data well if the goodness-of-fit indices met the following criteria: non-significant χ^2^-value (p ≥ 0.05) [35], ratio of χ^2^ and degrees of freedom (df) ≤ 2 [35], comparative fit index (CFI) and Tucker-Lewis index (TLI) > 0.95 [36], root mean square error of approximation (RMSEA) < 0.05 and narrow confidence interval (CI) for an excellent to close fit [37] (fair fit at values of 0.06–0.07, mediocre fit at values of 0.08–0.10, and poor fit at values above 0.10 [36]), and standardized root mean square residual (SRMR) < 0.05 [36]. Scaled measures were reported for all indices except SRMR (not available). Given the potential limitations of interpreting fit indices using the WLS estimator [38], we additionally reported fit indices obtained from the models using robust maximum likelihood (MLR).

Pearson correlation analyses were utilized to investigate convergent and divergent validity through correlations between the QOLIBRI-OS-KID/ADO total score and the questionnaires measuring symptom burden in the pTBI sample. We hypothesized strong positive correlations between the QOLIBRI-OS-KID/ADO and its long version and the PedsQL, assessing generic HRQoL, as evidence of convergence. Correlations between the GAD-7, the PHQ-9, and the PCSI-SR8/13 were anticipated to be small to medium and negative, indicating some overlap between the constructs and suggesting that higher symptom burden is associated with lower HRQoL and vice versa, but measuring different constructs as an evidence of divergence. Determination coefficient (R^2^) was computed to evaluate the amount of explained variance.

Hypotheses were tested using one-tailed t-tests in the pTBI sample. We presumed that girls, adolescents (aged 13–17 years), participants with inferior recovery (i.e., KOSCHI < 5b), and participants with greater symptom burden (i.e., GAD-7 and PHQ-9 scores > 4, and PCSI-SR8/13 scores ≥ M + 1SD), would report lower HRQoL than the boys, children (aged 8–12 years), participants with superior recovery, and those reporting no symptom burden, respectively (see Table 1, column “Cut-off applied” for more details on the cut-offs for the test instruments). These assumptions, also tested for the QOLIBRI-KID/ADO, were based on findings from previous studies [13]. Cohen’s d was calculated to determine effect size, with values of 0.20, 0.50, and 0.80 representing small, medium, and large effects, respectively [39]. Results were reported only for groups with n > 30.

Prior to establishing reference values, we conducted a multi-group confirmatory factor analyses (CFA) [40, 41] to compare HRQoL construct assessment between the pTBI and the general population sample and determine measurement invariance (MI). We estimated three models with increasing constraints and then compared them using the χ^2^ difference test. We first fitted the configural model, then assumed equality of thresholds, followed by equality of both thresholds and loadings. If the difference test yields a non-significant result (p ≥ 0.05), it suggests that the HRQoL construct is assessed equally in both groups and that the difference between samples is solely due to the HRQoL disparity. Thus, the QOLIBRI-OS-KID/ADO can be utilized in both general population and TBI samples, and providing reference values is appropriate. Furthermore, we estimated a multiple linear regression to identify significant interactions between gender, age, and presence of chronic health conditions and the QOLIBRI-OS-KID/ADO total score for possible stratification of the reference values (see Supplementary Material, Regression analyses).

Finally, the reference values were calculated using percentiles, allowing for a simple and straightforward interpretation of the patient’s scores in relation to a comparable general population without a history of pTBI. The distribution of QOLIBRI-OS-KID/ADO scores in the general population sample was divided into percentiles (i.e., 2.5%, 5%, 16%, 30%, 40%, 50%, 60%, 70%, 85%, 95%, and 97.5%), with values below the 16th percentile indicating below-average HRQoL [42] as compared to the general population and thus a clinically relevant impairment.

We used R version 4.3.0 [43] for all analyses, employing the packages Table 1 [44], *psych* [45], *lordif* [29], and *lavaan* [46]. Unless otherwise stated, the significance level was set at 5%.

## Results

### Participants

Overall, 297 children and adolescents (54.2% male; mean age 12.6 ± 2.67 years) after pTBI participated in the study. Most sustained a mild TBI (79.8%), with 7% having visible intracranial imaging abnormalities. Injury occurred on average 5.36 ± 2.70 years prior to study entry with falls being the most common cause (72.1%). Most participants had fully recovered (86.9%). The mean QOLIBRI-OS-KID/ADO score was 82.3 ± 13.7 points. A significant difference was observed when comparing HRQoL between participants surveyed in different settings (i.e., online vs. offline), with offline respondents (40.4% of the sample) being slightly more satisfied (ΔM = 3.19, d = 0.23, p = 0.049) than those interviewed online (59.6%). A subsample of n = 57 participants completed the QOLIBRI-OS-KID/ADO twice, with a mean test–retest interval of 12.12 ± 2.38 days.

Overall, 1997 children and adolescents (49.5% male; mean age 12.4 ± 2.85 years) from the German general population participated in the study. Most had no chronic health problems according to parental reports (87.5%). The mean QOLIBRI-OS-KID/ADO score was 77.6 ± 16.7 points. The HRQoL of the general population sample was significantly lower than that of the pTBI sample (ΔM = − 4.66, d = − 0.29, p < 0.001). When comparing the subgroup of participants from the general population sample without chronic health conditions to the pTBI sample, the effect decreased slightly but remained significant (ΔM = − 4.04, d = − 0.26, p < 0.001). For details, see Table 2.

**Table 2:** Sociodemographic and health-related data

Variable	Group/value	pTBI sample (N = 297)	General population sample (N = 1997)	p^d^
Sex/gender	Female	136 (45.8%)	1007 (50.4%)	0.151
	Male	161 (54.2%)	989 (49.5%)	
	Diverse	0 (0%)	1 (0.1%)	
Age (years)	M (SD)	12.6 (2.67)	12.4 (2.85)	0.135
	Mdn [Min, Max]	12.4 [8.00, 17.9]	12.0 [8.00, 17.0]	
Study setting^a^	Online	177 (59.6%)	1997 (100%)	N/A
	Offline	120 (40.4%)	–	
Education	None	3 (1.0%)	6 (0.3%)	N/A
	Pre-school	2 (0.7%)	–	
	Primary school	78 (26.3%)	556 (27.8%)	
	Special school	2 (0.7%)	83 (4.2%)	
	Vocational school	–	78 (3.9%)	
	Secondary school	7 (2.4%)	119 (6.0%)	
	Secondary/middle school	41 (13.8%)	533 (26.7%)	
	Preparatory high school	–	592 (29.6%)	
	High school	155 (52.2%)	–	
	Missing or not assignable	9 (3.0%)	30 (1.5%)	
Injury cause	Traffic accident	31 (10.4%)	–	N/A
	Fall	214 (72.1%)	–	
	Collision with persons/objects (other than vehicles)	44 (14.8%)	–	
	Violence/suicide attempt	2 (0.7%)	–	
	Other cause	5 (1.7%)	–	
	Missing	1 (0.3%)	–	
Chronic health conditions^b^	Yes	43 (14.5%)	249 (12.5%)	
	No	253 (85.2%)	1748 (87.5%)	
	Missing	1 (0.3%)	–	
TBI severity	Mild	237 (79.8%)	–	N/A
	Moderate	30 (10.1%)	–	
	Severe	30 (10.1%)	–	
Cerebral Lesion(s) found in neuroimaging^c^	Yes	73 (24.6%)	–	N/A
	No	224 (75.4%)	–	
	Missing	0 (0%)	–	
Recovery (KOSCHI)	4b	15 (5.1%)	–	N/A
	5a	21 (7.1%)	–	
	5b	258 (86.9%)	–	
	Missing	3 (1.0%)	–	
Time since injury in years	M (SD)	5.36 (2.70)	–	N/A
	Mdn [Min, Max]	5.29 [0.457, 9.95]	–	
Head-/aches, Physical impairments, or mental health problems after TBI	Yes	145 (48.8%)	–	N/A
	No	150 (50.5%)	–	
	Missing	2 (0.7%)	–	
QOLIBRI-OS-KID/ADO (Self-report)	Mean (SD)	82.3 (13.7)	77.6 (16.7)	
	Mdn [Min, Max]	83.3 [20.8, 100]	79.2 [0, 100]	** < 0.001**
Learning rate (RAVLT)	Below average (≤ M − 1SD)	57 (19.2%)	–	N/A
	Average	174 (58.6%)	–	
	Above average (≥ M + 1SD)	65 (21.9%)	–	
	Missing	1 (0.3%)	–	
GAD-7 (Proxy-report)	No depression (0)	45 (15.2%)	–	N/A
	Minimal depression (1–4)	164 (55.2%)	–	
	Mild to severe depression (≥ 5)	87 (29.3%)	–	
	Missing	1 (0.3%)	–	
PHQ-9 (Proxy-report)	No depression (0)	33 (11.1%)	–	N/A
	Minimal depression (1–4)	175 (58.9%)	–	
	Mild to severe depression (≥ 5)	88 (29.6%)	–	
	Missing	1 (0.3%)	–	
PCSI-SR8/13 (Self-report)	Below average (≤ M − 1SD)	26 (8.8%)	–	N/A
	Average	221 (74.4%)	–	
	Above average (≥ M + 1SD)	47 (15.8%)	–	
	Missing	3 (1.0%)	–	

* –* information is not relevant for this sample, *N* absolute frequencies, *%* relative frequencies, *M* mean, *SD* standard deviation, *Mdn* median, *Min* minimum, *Max* maximum, *TBI* traumatic brain injury, *KOSCHI* King’s outcome scale for closed head injury, *RAVLT* Rey auditory learning test, *GAD-7* generalized anxiety disorder 7, *PHQ-9* patient health questionnaire 9, *PCSI-SR* postconcussion symptom inventory—self report version (*SR8* children aged 8–12 years, *SR13* adolescents aged 13–18 years)

^a^The study setting implies a face-to-face interview, either online or offline (pTBI sample), or an online survey (general population sample)

^b^Chronic health conditions were assessed as present (yes) and absent (no) in the TBI sample as at least one (yes) and none (no) in the general population sample

^c^No imaging was performed in 162 cases (54.0%). These cases were classified as “no lesion” because there was no medical indication for a lesion

^d^p-values are obtained from either a *t*-test for continuous variables or a two-dimensional chi-squared test for categorical variables. N/A: analyses are not performed either because of small numbers of cases in some categories (n < 5) or because the information is not available for both samples. Values in bold are significant at 5%

### Item characteristics, reliability, and DIF analyses

At the item level, participants in both samples tended to indicate a rather high level of HRQoL, as the mean scores per item were around 4, corresponding to the response category “Quite (satisfied)”. As a result, the items were moderately to highly skewed.

Reliability coefficients were α = 0.75 and ω = 0.81 (pTBI sample) and α = 0.85 and ω = 0.86 (general population sample). The exclusion of none of the items increased the initial Cronbach’s α of the scale. The CITCs were > 0.40 in both samples. The DIF analyses suggested no meaningful differences in the items between children and adolescents. Although two items (i.e., “Autonomy” and “Future Prospects”) exhibited significant p-values in the general population sample, McFadden’s R^2^ values were less than 0.01, suggesting that these differences were negligible. The QOLIBRI-OS-KID/ADO data from children and adolescents were considered acceptable for joint analyses. For details, see Table 3.

**Table 3:** Item characteristics and results of reliability and DIF analyses

		Item responses and characteristics							Reliability				DIF analyses	
Sample	Item	Not at all	Slightly	Moderately	Quite	Very	M (SD)	SK	Cronbach’s αª	McDonald’s ω	α if item omittedª	CITC	p	McFadden’s R^2^
pTBI Sample	Physical problems	2 (0.7%)	6 (2.0%)	17 (5.7%)	84 (28.3%)	188 (63.3%)	4.52 (0.75)	− 1.82	0.75	0.81	0.39	0.47	0.013	0.016
	Cognition	2 (0.7%)	15 (5.1%)	53 (17.8%)	131 (44.1%)	96 (32.3%)	4.02 (0.88)	− 0.74			0.56	0.65	0.865	< 0.001
	Emotions	5 (1.7%)	20 (6.7%)	62 (20.9%)	128 (43.1%)	82 (27.6%)	3.88 (0.95)	− 0.72			0.55	0.63	0.577	0.001
	Autonomy	1 (0.3%)	2 (0.7%)	17 (5.7%)	45 (15.2%)	232 (78.1%)	4.70 (0.64)	− 2.38			0.42	0.49	0.029	0.017
	Social aspects	1 (0.3%)	5 (1.7%)	32 (10.8%)	102 (34.3%)	157 (52.9%)	4.38 (0.77)	− 1.15			0.49	0.55	0.290	0.004
	Future prospects	5 (1.7%)	9 (3.0%)	34 (11.4%)	109 (36.7%)	140 (47.1%)	4.25 (0.89)	− 1.32			0.55	0.63	0.813	0.001
General population sample	Physical problems	17 (0.9%)	75 (3.8%)	275 (13.8%)	657 (32.9%)	973 (48.7%)	4.20 (0.89)	− 1.12	0.85	0.86	0.83	0.62	0.011	0.002
	Cognition	31 (1.6%)	106 (5.3%)	375 (18.8%)	854 (42.8%)	631 (31.6%)	4.00 (0.92)	− 0.83			0.82	0.63	0.012	0.002
	Emotions	31 (1.6%)	93 (4.7%)	411 (20.6%)	886 (44.4%)	576 (28.8%)	3.90 (0.9)	− 0.77			0.81	0.68	0.394	< 0.001
	Autonomy	13 (0.7%)	44 (2.2%)	228 (11.4%)	647 (32.4%)	1065 (53.3%)	4.40 (0.82)	− 1.26			0.83	0.60	< 0.001	0.004
	Social aspects	25 (1.3%)	80 (4.0%)	326 (16.3%)	807 (40.4%)	759 (38.0%)	4.10 (0.90)	− 0.94			0.83	0.60	0.078	0.001
	Future prospects	29 (1.5%)	67 (3.4%)	378 (18.9%)	918 (46.0%)	605 (30.3%)	4.00 (0.87)	− 0.84			0.82	0.66	< 0.001	0.003

*M* mean, *SD* standard deviation, *SK* skewness, *CITC* corrected item-total correlations, *p* p-value, *DIF* differential item functioning between children (8–12 years) and adolescents (13–17 years)

ªStandardized α coefficients were used

The test–retest reliability in the pTBI sample was ICC(2,1) = 0.68, CI_95%_ [0.51, 0.80], with a SEm of 6.35 points. A change of MDC = 17.59 points in the total score was considered a “true” change in HRQoL.

### Validity

**Fig. 2:**
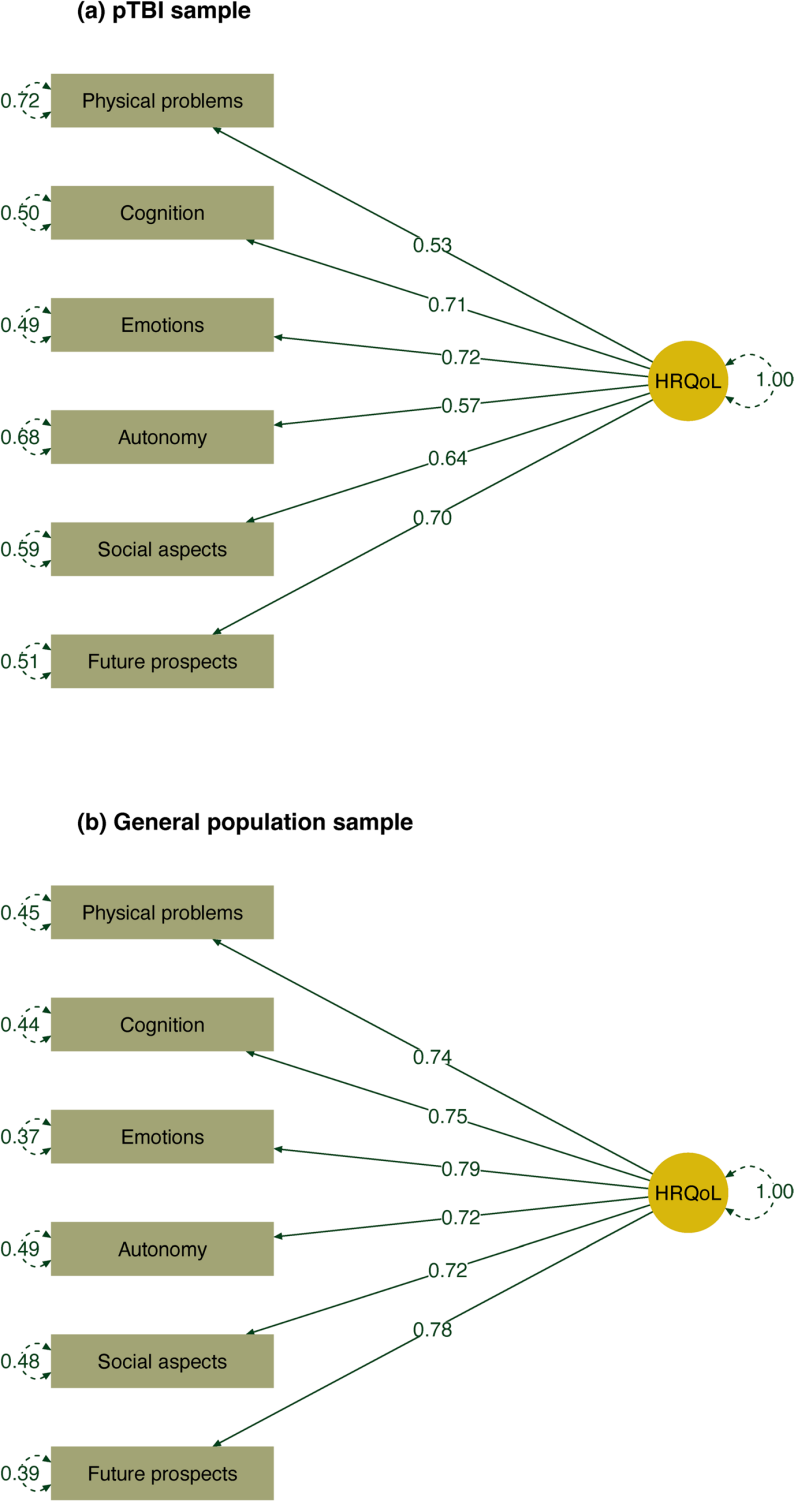
Results of the CFA (standardized parameter estimates; model using the WLS estimator) for **a** the pTBI sample and **b** for the general population sample

The one-dimensional factorial structure showed a good fit in both samples, with most indices being within acceptable ranges. The scaled fit statistics for the pTBI sample from the model using the WLS estimator were χ^2^(9) = 20.01, p = 0.018, χ^2^/df = 2.22, CFI = 0.98, TLI = 0.97, RMSEA[90% CI] 0.06 [0.03, 0.10], and SRMR = 0.05. The robust fit measures additionally derived from the model using the MLR estimator were CFI = 0.96, TLI = 0.94, RMSEA[90% CI] 0.07 [0.03, 0.11], SRMR = 0.04. The scaled fit statistics for the general population sample were χ^2^(9) = 119.77, p < 0.001, χ^2^/df = 13.31, CFI = 0.99, TLI = 0.98, RMSEA[90% CI] 0.08 [0.07, 0.09], and SRMR = 0.03. Robust fit measures were CFI = 0.98, TLI = 0.97, RMSEA[90% CI] 0.07 [0.05, 0.09], SRMR = 0.03 Fig. 2 provides an overview on standardized model parameters for the models using the WLS estimator.

Convergent validity analyses showed that the correlation coefficients between the QOLIBRI-OS-KID/ADO and the QOLIBRI-KID/ADO ranged from 0.45 (Emotions scale) to 0.82 (Total score). The respective amount of variance explained varied from 20% to 67%. The association with the PedsQL measuring generic HRQoL ranged from 0.52 (Social Functioning) to 0.74 (Total score), with variance explained between 27% and 55%, respectively.

Divergent validity analyses results revealed hypothesized negative associations between the QOLIBRI-OS-KID/ADO total score and instruments measuring symptom burden (i.e., GAD-7, PHQ-9, and PCSI-SR8/SR13) in the pTBI sample. The coefficients ranged from -0.29 (anxiety) to -0.56 (post-concussion symptoms), explaining 8% to 31% of the variability of the scores. For details, see Table 4.

**Table 4:** Pearson correlations between the QOLIBRI-OS-KID/ADO total scores and other instruments

Instrument	Scale	n	r	R^2^ (%)
QOLIBRI-KID/ADO	Cognition	297	0.69	48
	Self	295	0.75	56
	Daily life & autonomy	297	0.76	58
	Social relationships	297	0.71	50
	Emotions	297	0.45	20
	Physical problems	297	0.50	25
	Total score	295	0.82	67
PedsQL	Emotional functioning	297	0.60	36
	Social functioning	296	0.52	27
	School functioning	296	0.65	42
	Physical functioning	296	0.73	53
	Psychosocial functioning	297	0.60	36
	Total score	296	0.74	55
GAD-7 (Proxy)	Total score	296	− 0.29	8
PHQ-9 (Proxy)	Total score	296	− 0.43	18
PCSI-SR8/SR13	Total score	294	− 0.56	31

*n* absolute frequencies, *r* Pearson correlation coefficient (all p < 0.001), *R*^2^ determination coefficient, *QOLIBRI-KID/ADO* quality of life after injury—children and adolescents, *PedsQL* pediatric quality of life inventory, *GAD-7* generalized anxiety disorder 7, *PHQ-9* patient health questionnaire 9, *PCSI-SR8/SR13* postconcussion symptoms inventory (*SR8* children aged 8–12 years, *SR13* adolescents: aged 13–18 years)

Adolescents (ΔM = − 5.26, d = − 0.39, p < 0.001), participants with parent-reported post-pTBI health problems (ΔM = − 5.21, d = − 0.39, p < 0.001), those with higher levels of anxiety (ΔM = − 6.88, d = − 0.51, p < 0.001), depression (ΔM = − 8.77, d = − 0.67, p < 0.001), and post-concussion symptoms (ΔM = − 15.55, d = − 1.30, p < 0.001) had significantly lower HRQoL compared to the corresponding reference groups. All other comparisons were not significant or did not reach a sufficient number of individuals in at least one group (Table 5).

**Table 5:** Results of hypothesis testing between predefined groups for the QOLIBRI-OS-KID/ADO total score in the TBI sample

Variable	Group	n	M	SD	t	df	p	d
Age group	Adolescents	131	79.33	15.02	− 3.34	295	** < 0.001**	− 0.39
	Children	166	84.59	12.12				
Sex	Female	136	81.28	14.50	− 1.14	295	0.127	− 0.13
	Male	161	83.10	12.98				
Time since injury	< 4 Years	99	80.85	14.51	− 1.26	295	0.104	− 0.16
	4–10 Years	198	82.98	13.26				
TBI severity	Moderate-to-severe TBI	60	81.39	15.79	− 0.56	295	0.290	− 0.08
	Mild TBI	237	82.49	13.15				
Recovery (KOSCHI)*	Incomplete recovery (KOSCHI < 5)	15	70.83	19.92	–	–	–	–
	Good recovery (KOSCHI = 5)	279	82.86	13.07				
(Head)aches, physical impairment, or mental health problems after TBI	Yes	145	79.54	15.11	− 3.32	293	**0.001**	− 0.39
	No	150	84.75	11.68				
Learning rate (RAVLT)	Below average (≤ M − 1SD)	57	82.16	15.20	− 0.03	294	0.489	< 0.01
	Average and above	239	82.22	13.33				
GAD-7 (Proxy-report)	Mild to severe anxiety (> 4)	87	77.39	15.79	− 4.03	294	** < 0.001**	− 0.51
	No to minimal anxiety (0–4)	209	84.27	12.25				
PHQ-9 (Proxy-report)	Mild to severe depression (> 4)	88	76.09	16.10	− 5.25	294	** < 0.001**	− 0.67
	No to minimal depression (0–4)	208	84.86	11.68				
PCSI-SR8/-SR13 (Self-report)	Above average (≥ M + 1SD)	47	69.50	14.31	− 8.17	292	** < 0.001**	− 1.30
	Average and less	247	85.05	11.47				

*n* absolute frequencies, *M* mean, *SD* standard deviation, *t* t-value, *df* degrees of freedom, *p* p-value, *d* Cohen’s d (values of 0.20, 0.50, and 0.80 correspond to a small, medium, and large effects, respectively), *QOLIBRI-KID/ADO* quality of life after injury—children and adolescents, *PedsQL* pediatric quality of life inventory, *RAVLT* Rey auditory learning test, *GAD-7* generalized anxiety disorder 7, *PHQ-9* patient health questionnaire 9, *PCSI-SR8/SR13* postconcussion symptoms inventory (*SR8* children aged 8–12 years, *SR13* adolescents aged 13–18 years), p-values in bold are significant at 5%

*t-tests conducted for groups with n > 30

### Measurement invariance

The results of the MI analyses showed no significant differences between the models with increasing restrictions (p > 0.05), suggesting that the construct of HRQoL was measured equally in both samples. In view of these results, it seems appropriate to use the data obtained from the general population sample as a reference for the QOLIBRI-OS-KID/ADO. For details, see Table 6.

**Table 6:** Results of measurement invariance testing (general population sample vs. TBI sample)

Constraints	χ^2^ (df)	*p*	χ^2^/df	CFI	TLI	RMSEA [90% CI]	SRMR	χ^2^ (df)	Δ χ^2^	Δ df	*p*
Baseline	123.17 (18)	< 0.001	6.84	**0.99**	**0.98**	**0.071 [0.060, 0.084]**	**0.031**	67.176 (18)	–	–	–
Thresholds	123.13 (30)	< 0.001	4.10	**0.99**	**0.99**	**0.052 [0.043, 0.062]**	**0.031**	72.090 (30)	7.759	12	**0.804**
Thresholds and loadings	108.35 (35)	< 0.001	3.10	**0.99**	**0.99**	**0.043 [0.034, 0.052]**	**0.032**	76.960 (35)	5.248	5	**0.386**

*χ*^2^ scaled chi-square statistics, *df* scaled degrees of freedom, *p* p-value, *χ*^*2*^*/df* scaled ratio (cut-off: ≤ 2), *CFI* scaled comparative fit index (cut-off: > 0.90), *TLI* scaled Tucker-Lewis Index (cut-off: > 0.95), *RMSEA [90% CI]* scaled root mean square error of approximation with 90% confidence interval (cut-off: < 0.06), *SRMR* scaled standardized root mean square residual (cut-off: < 0.08), *∆χ*^2^ change in chi square values between compared models, *∆df* change in degrees of freedom between compared models, *∆CFI* change in CFI between compared models (cut-off: < 0.01), *∆RMSEA* change in RMSEA between compared models (cut-off: ≤ 0.01)

Values in bold indicate at least a satisfactory/mediocre model fit according to the respective cut-offs and/or are within an acceptable range

### Reference values

Based on the results of the regression analyses, the reference values were further stratified by gender and age, but excluding those sustaining chronic health conditions due to insufficient number of cases (see Supplementary Material, Regression analyses and Table S2). Table 7 provides reference values for the QOLIBRI-OS-KID/ADO total score. The following example illustrates the application of the reference values. A 15-year-old female patient has a QOLIBRI-OS-KID/ADO total score of 60. This means that her score is between the 5% and 16% percentiles, and she is in the under-average clinically relevant HRQoL range. Accordingly, compared to girls aged 13–17 from the general population, her HRQoL is below average and there is an indication for further diagnostics. In this case, a longer version of the instrument could be used to identify HRQoL domains that are particularly affected (e.g., cognition or emotions). Depending on the results, further diagnostics, such as neuropsychological testing or screening for depression or anxiety, may be performed to gain more information about what may have negatively impacted the patient’s HRQoL. If the QOLIBRI-OS-KID/ADO total score is in the normal range, there is no indication for further diagnostics.

**Table 7:** Reference values for the QOLIBRI-OS-KID/ADO from the general population sample stratified by gender and age

			Low HRQoL		− 1 SD			Mdn			+ 1 SD		High HRQoL
Gender	Age	N	2.5%	5%	16%	30%	40%	50%	60%	70%	85%	95%	97.5%
Male	8–12	462	48	50	66	71	75	79	83	88	96	100	100
	13–17	401	42	50	67	75	79	83	83	88	96	100	100
Female	8–12	463	46	50	67	75	75	83	83	88	96	100	100
	13–17	422	40	50	62	71	75	79	83	88	96	100	100
Total sample	8–17	1748	42	50	62	71	75	79	83	88	96	100	100

Only two gender groups (male and female) are included in the analyses due to the small number of diverse participants (n = 1). Participants with chronic health conditions (n = 249) were not included because the number of cases was insufficient to provide stratified reference values

50% percentiles represent 50% of the distribution corresponding to both the median (Mdn) and the mean; SD: standard deviation; values from − 1 standard deviation (16%; rounded to the next integer) to + 1 standard deviation (85%; rounded to the next integer) are within the normal range; values below 16% indicate low HRQoL and values above 85% indicate high HRQoL

Another interpretation would be to determine a cut-off for the assessment of clinical relevance. In this case, QOLIBRI-OS-KID/ADO scores less than or equal to 62 indicate impaired HRQoL in females aged 13–17 years. The score of 89 from our example is above the cut-off of 62, indicating that the patient did not report clinically relevant impairment.

An interactive app for the reference values can be found online (https://reference-values.shinyapps.io/Tables_Reference_values/, tab QOLIBRI-OS-KID/ADO; last access on 05.07.2023).

## Discussion

The aim of this study was to investigate the psychometric properties of the QOLIBRI-OS-KID/ADO, a short scale assessing disease-specific HRQoL after pTBI, and to provide reference values from a general population sample. Based on the results of the psychometric analyses, we recommend the use of the QOLIBRI-OS-KID/ADO, especially when a brief overview of TBI-specific HRQoL is needed. The established reference values serve as a basis for screening decisions (e.g., further administration of a longer version of the instrument to gain more detailed insight into impaired HRQoL domains or omission of further steps in the absence of an indication) and provide information on the degree of overall HRQoL impairment after pTBI. Some of the central findings are discussed below.

The reliability of the QOLIBRI-OS-KID/ADO was satisfactory, indicating that the items are internally consistent, and the test results are repeatable within a period of 20 days. A score change of approximately 18 points represents a true change in HRQoL between two measurement occasions. Future studies should further focus on the clinically meaningful change to evaluate the course of recovery and the effectiveness of therapies and treatments after pTBI.

The DIF analyses suggested that the instrument is applicable to children and adolescents, allowing for aggregated analyses, item-level comparison, and longitudinal HRQoL monitoring. The psychometric properties of the QOLIBRI-OS-KID/ADO are similar to those of the QOLIBRI-KID/ADO [13], and the correlation between the total scores indicates strong construct overlap. The overlap with generic HRQoL as measured by the PedsQL was slightly lower. Similar associations between generic and disease-specific HRQoL measures have also been found for other pediatric health conditions (e.g., allergies [47], renal disease [48], or epilepsy [49]). This finding indicates that the constructs are related—also in terms of convergent validity—but not completely identical, suggesting that generic HRQoL measures cannot serve as substitutes for disease-specific PROMs.

Negative associations between the QOLIBRI-OS-KID/ADO and the instruments measuring symptom burden suggest that higher burden is associated with reduced disease-specific HRQoL and vice versa, which is consistent with findings for the QOLIBRI-KID/ADO [13, 14]. Finally, the goodness of fit of the one-factor model indicates that the assessment of HRQoL occurs unidimensionally and that the use of the QOLIBRI-OS-KID/ADO total score is reasonable. The use of both WLS and ML estimators in the CFA analyses supports this finding.

The QOLIBRI-OS-KID/ADO covers the HRQoL domains specified in the 35-item form in a general way and may therefore be less able to detect all differences between predefined sociodemographic and clinical groups than the long version. This may explain why no differences in sex, time since injury, and TBI severity were observed in the known group analyses in the pTBI sample. However, significant differences between age groups and between children and adolescents with and without post-injury health problems indicate that, as hypothesized, adolescents and those reporting health problems report lower HRQoL than comparison groups. This finding suggests that the QOLIBRI-OS-KID/ADO reflects the ability of the 35-item version to discriminate between predefined groups [13]. In addition, significantly lower QOLIBRI-OS-KID/ADO scores were observed in children and adolescents with higher levels of symptom burden compared to the low symptom burden groups. This reflects the findings from the previous studies suggesting decreased overall HRQoL in children with post-pTBI affective disorders [50] and post-concussion symptoms in both the acute [51] and post-acute phases [52]. Similar to the results of the long version [14], we did not observe significant differences in HRQoL between mild and moderate-to-severe pTBI groups. This finding may be explained by the small number of children and adolescents with greater TBI severity, which also reflects the prevalence of more severe TBI in pediatric populations [53], and thus lower power to detect the differences. Given that the adult version of the instrument showed a clear ability to discriminate between different groups of TBI severity at three, six, and 12 months after injury in a large pan-European study [17], further evidence is warranted to support the utility of the QOLIBRI-OS-KID/ADO across all severity levels in children and adolescents.

We found significantly lower HRQoL in the general population sample compared to the pTBI sample, although the effect was small. This may seem surprising at first, but this finding can be further explained. First, the pTBI sample included children and adolescents in the post-acute phase after mild injury. Therefore, the children and adolescents may have mostly recovered from the injury and/or adapted to its consequences, also in terms of HRQoL [54]. Second, by removing the reference to a specific health condition, as was done in adapting the QOLIBRI-OS-KID/ADO for administration to the general population, a broader or different interpretation of life and health domains to which responses can be related may be possible, resulting in greater variability in responses. Finally, due to the large size of the general population sample, the test may have been overpowered, detecting small effects that may be less relevant from a content-related perspective. In fact, the average difference between the samples was 4 points on a scale of 0–100. Future analyses using a matched sample adjusted for age, sex, and presence of chronic health conditions may shed more light on these differences.

Irrespective of gender and age, a cut-off of 62 on the QOLIBRI-OS-KID/ADO total score can be considered clinically relevant. For boys aged 8–12 years and 13–17 years, scores below 66 and 67, respectively, indicate impaired HRQoL, and for girls in both age groups, 67 and 62, respectively. With these scores in mind, possible deficits can be identified, and more detailed and personalized diagnostics can be applied to better understand which HRQoL domains are affected and need therapeutic interventions. Compared to the previously established cut-off value of 52 for the adult version of the questionnaire [55], the critical HRQoL score in children and adolescents is higher. However, the adult cut-off value was methodologically derived from the norm-based Mental Component Summary score of the Short Form Survey [56] using regression analyses and not from the comparable general adult population sample.

### Strengths and limitations

The main strength of this study is that it provides a first short scale assessing disease-specific HRQoL after pTBI in the German-speaking language context, complemented by reference values obtained from a German general population. The study fills the gap of economic PROMs and provides interpretation guidelines in the field of pTBI. However, some limitations should be mentioned.

Overall, the number of children and adolescents after pTBI enrolled in the study represents 9% of the total number of invitations sent. Participation in the study was voluntary, and the decision to enroll should have been made by the parents. For privacy reasons, we were unable to perform analyses on known characteristics between those who enrolled and those who did not. Therefore, we can only assume that there may have been several reasons for non-response (ranging from change of address due to relocation, unwillingness to participate, or concerns about re-traumatizing the children through repeated explicit confrontation with the traumatic event). However, the planned number of participants in the project has been reached [14]. These issues have been addressed in more detail elsewhere [14].

The children and adolescents included in the present study mostly suffered from mild pTBI (ca. 80%) and were in the post-acute stage of injury (5.36 ± 2.70 years). Therefore, further validation in a more acute sample and in moderate to severe pTBI is warranted. The same applies to the levels of recovery, which could not be compared due to the small number of cases in the less favorable recovery groups: good recovery (KOSCHI 5a: 7.1%) and upper moderate disability (KOSCHI 4b: 5.1%). Further research is needed to better understand the applicability of the QOLIBRI-OS-KID/ADO total score to differentiate between different levels of recovery/disability.

Furthermore, the significant difference in HRQoL between online and offline interviewed patients can be explained by the higher prevalence of children and adolescents with pTBI within the first year (8.4% vs. 0%) and of moderate and severe TBI (23.4% vs. 14.8%) in the online group.

The general population sample was recruited online, which has both advantages and disadvantages. The main advantage is the accessibility of the relatively large number of participants. The potential disadvantages are a certain bias (e.g., recruitment of individuals with Internet access), non-standardized setting of data collection, and difficulty in verifying individuals [57]. Nevertheless, we have tried to maintain data quality by recruiting participants from internationally recognized market research agencies, controlling for inconsistent responses, and excluding “speeders” from the data set. While the planned number of cases of N = 2000 was almost reached also after data cleaning, the number of survey starts that ended with a termination was 61% (response rate 39%), indicating a possible selection bias and that only those interested completed the questionnaires. However, comparative analyses between respondents and non-respondents were not possible because the research agencies were not allowed to disclose information about those who had withdrawn.

The subsample with chronic health conditions was not sufficient to stratify the reference values. Therefore, the reference values provided represent the ideal norm. Expanding the reference values with information from subgroups with chronic health conditions would provide additional insight into HRQoL in the general population and allow for more nuanced comparisons with pTBI patients.

## Conclusions

The QOLIBRI-OS-KID/ADO is a short, reliable, and valid PROM assessing disease-specific HRQoL after pTBI. Combined with reference values obtained from a general population sample, it provides a cost-effective screening for clinical practice and research. Future studies should focus on its applicability in more acute and severe samples and on the long-term validity, especially in combination with the adult version, which would provide further insight into the longitudinal effects of pTBI. Finally, translation into other languages would allow multinational studies to fill the knowledge gap regarding pTBI-specific HRQoL internationally.

## Supplementary Information

Below is the link to the electronic supplementary material.

Supplementary file1 (DOCX 33 KB)

## Data Availability

The data presented in this study are available on reasonable request from the corresponding author. Data are not publicly available for privacy reasons.

## Abbreviations

CFA: Confirmatory factory analysis
CFI: Comparative fit index
CI: Confidence interval
CITC: Corrected item-total correlations
DIF: Differential item functioning
GAD-7: Generalized anxiety disorder 7
HRQoL: Health-related quality of life
LORDIF: Logistic ordinal regression differential item functioning
MDC: Minimal detectable change
MI: Measurement invariance
PCSI-SR: Postconcussion inventory—self report
PedsQL: Pediatric quality of life inventory
PHQ-9: Patient health questionnaire 9
PROM: Patient-reported outcome measure
pTBI: Pediatric traumatic brain injury
QOLIBRI-KID/ADO: Quality of life after brain injury for children and adolescents
OLIBRI-OS-KID/ADO: Quality of life after brain injury—overall scale for children and adolescents
RAVLT: Rey auditory verbal learning test
RMSEA: Root mean square error of approximation
SEm: Standard error of measurement
SRMR: Standardized root mean square residual
TBI: Traumatic brain injury
TLI: Tucker-Lewis index
