# Correction: A short scale to measure health-related quality of life after traumatic brain injury in children and adolescents (QOLIBRI-OS-KID/ADO): psychometric properties and German reference values

**DOI:** 10.1007/s11136-024-03845-3

**Published:** 2024-12-03

**Authors:** Marina Zeldovich, Leonie Krol, Inga K. Koerte, Katrin Cunitz, Matthias Kieslich, Marlene Henrich, Knut Brockmann, Anna Buchheim, Michael Lendt, Christian Auer, Axel Neu, Joenna Driemeyer, Ulrike Wartemann, Claudius Thomé, Daniel Pinggera, Steffen Berweck, Michaela V. Bonfert, Joachim Suss, Holger Muehlan, Nicole von Steinbuechel

**Affiliations:** 1 Institute of Psychology, Faculty of Psychology and Sport Science, University of Innsbruck, Innsbruck, Austria; 2 Faculty of Psychotherapy Science, Sigmund Freud University, Vienna, Austria; 3 Department of Psychology, Clinical Psychology and Psychotherapy, Philipps University of Marburg, Marburg, Germany; 4 cBRAIN / Department of Child and Adolescent Psychiatry, Psychosomatics, and Psychotherapy, LMU University Hospital, Ludwig-Maximilian University, Munich, Germany; 5 Psychiatry Neuroimaging Laboratory, Department of Psychiatry, Mass General Brigham, Boston, MA USA; 6 Department of Psychiatry and Psychotherapy, University Medical Center Goettingen, Goettingen, Germany; 7 Department of Paediatric Neurology, Hospital of Goethe University, Frankfurt, Germany; 8 Interdisciplinary Pediatric Center for Children with Developmental Disabilities and Severe Chronic Disorders, Department of Pediatrics and Adolescent Medicine, University Medical Center, Goettingen, Germany; 9 Department of Neuropediatrics, St. Mauritius Therapeutic Clinic, Meerbusch, Germany; 10 Department of Neurosurgery, Kepler University Hospital GmbH, Johannes Kepler University Linz, Linz, Austria; 11 Clinical Research Institute für Neurosciences, Faculty of Medicine, Johannes Kepler University Linz, Linz, Austria; 12 Department of Neurology and Neuropediatry, VAMED Klinik Geesthacht GmbH, Geesthacht, Germany; 13 Department of Pediatrics, University of Hamburg-Eppendorf, Hamburg, Germany; 14 Department of Neuropediatrics, VAMED Klinik Hohenstücken GmbH, Brandenburg an der Havel, Germany; 15 Department of Neurosurgery, Medical University lnnsbruck, Innsbruck, Austria; 16 Specialist Center for Paediatric Neurology, Neurorehabilitation and Epileptology, Schoen Klinik, Vogtareuth, Germany; 17 Department of Pediatric Neurology and Developmental Medicine and LMU Center for Development and Children with Medical Complexity, Dr. Von Hauner Children’s Hospital, LMU University Hospital, Munich, Germany; 18 Department of Pediatric Surgery, Wilhelmstift Catholic Children’s Hospital, Hamburg, Germany; 19 Department of Health and Prevention, University of Greifswald, Greifswald, Germany

**Quality of Life Research (2024) 33:3039-3056**

10.1007/s11136-024-03764-3

In the original published article, the sentence “The QOLIBRI-OS-KID/ADO was highly correlated with its long version (R2 = 67%) and showed an overlap with disease-specific HRQoL (R2 = 55%) in the TBI sample.” appearing in the results subsection of **Abstract** should have been “The QOLIBRI-OS-KID/ADO was highly correlated with its long version (R2 = 67%) and showed an overlap with generic HRQoL (R2 = 55%) in the TBI sample.”

The original article has been corrected.

