# Supplementary material for: A short scale to measure health-related quality of life after traumatic brain injury in children and adolescents (QOLIBRI-OS-KID/ADO): psychometric properties and German reference values
Source: Qual Life Res. 2024 Aug 31;33(11):3039–56. doi: 10.1007/s11136-024-03764-3 (PMC11541294; doi:10.1007/s11136-024-03764-3)
Supplement: Supplementary file 1 — Supplementary file1 (DOCX 33 KB) [file 11136_2024_3764_MOESM1_ESM.docx]

# Supplemental Material

## Development of the QOLIBRI-OS-KID/ADO

The development of the QOLIBRI-OS-KID/ADO was based on the adult version of the QOLIBRI-OS [1], with the wording adapted for children. The first version of the instrument was subjected to cognitive debriefing. For this purpose, seven children and adolescents (three after TBI and four without a history of TBI) completed the questionnaire while discussing their understanding of the instructions, items, and item responses aloud with the examiner. Participants in the cognitive debriefing had the opportunity to provide feedback on the comprehensibility of the content and to suggest rephrasing. This led to a change in the wording of the instructions (“overall” satisfied (German: “im Ganzen”) instead of “in total” satisfied (German: “insgesamt”) and to addressing the children directly with an additional sentence about the aim of the questionnaire (“This is why we would like to know how you feel afterwards [after the head injury].”). Furthermore, we added two examples to explain the content of two questions.

**Supplemental Table S1** Final version of the instructions and items of the QOLIBRI-OS-KID/ADO.

| **Instruction** | |  |
| --- | --- | --- |
| **Original German version** | **English translation**^a^ |  |
| Dieser Fragebogen ist für Kinder und Jugendliche nach einem Unfall/einer Kopfverletzung gedacht. Deswegen möchten wir wissen, wie es Dir danach geht. Wie zufrieden bist Du im Ganzen mit etwas, wenn Du an jetzt und an die letzte Woche denkst. | This questionnaire is for children and adolescents after an accident / a brain injury. We would like to know how satisfied you are, overall, with things when you are thinking about now and the past week. |  |
|  |  |  |
|  |  |  |
|  |  |  |
|  |  |  |
|  |  |  |
| **Items** | |  |
| **Original German version** | **English translation**^a^ |  |
| Wie zufrieden bist Du im Ganzen damit, wie es Dir körperlich geht, zum Beispiel, wie Du Dich bewegen kannst? | Overall, how satisfied are you with your body, for example, how you can move? |  |
| Wie zufrieden bist Du im Ganzen damit, wie Du denken, Dich konzentrieren und erinnern kannst? | Overall, how satisfied are you with how you can think, concentrate, and remember? |  |
| Wie zufrieden bist Du im Ganzen mit Deinen Gefühlen (z.B. fröhlich, glücklich, traurig, wütend zu sein)? | Overall, how satisfied are you with your feelings (e.g., feeling happy, sad, angry)? |  |
| Wie zufrieden bist Du im Ganzen damit, Dinge alleine zu schaffen, die Du jeden Tag machst, zum Beispiel Dich anzuziehen? | Overall, how satisfied are you with how you do everyday things without help, for example, getting dressed? |  |
| Wie zufrieden bist Du im Ganzen mit Deinen Freunden und wie Du Deine Zeit nach der Schule verbringst? | Overall, how satisfied are you with your friendships and how you spend your time after school? |  |
| Wie zufrieden bist Du im Ganzen mit Deinem Leben und damit, wie Du Dir Deine Zukunft vorstellst? | Overall, how satisfied are you with your life right now and how you imagine your future? |  |

^a^ The English translation is not yet validated and is for informational purposes only.

## Regression analyses

To adjust for potential stratification of the reference values based on significant differences in certain groups of individuals from the general population sample, we conducted a multiple linear regression analysis using QOLIBRI-OS-KID/ADO as the outcome and gender, age, and the presence of chronic health conditions as the independent variables. Additionally, we included second-order interactions (e.g., gender*age) in the model equation. For significant factor levels (i.e., p < 0.05), reference values needed to be separately provided.

The results of the regression analyses indicated a significant influence of selected factors and their interactions on the QOLIBRI-OS-KID/ADO total score (see Supplemental Table S2). Only two gender groups (male and female) were included in the analyses due to the small number of diverse participants (n = 1). In particular, age, presence of chronic health complaints and interactions between age and gender and age and presence of chronic health complaints were significant (p < 0.05). Therefore, a stratification of the reference values based on these variables was indicated. However, due to the relatively small number of children and adolescents with chronic conditions (n = 249), we could not provide reliable reference information. We therefore decided to calculate percentiles excluding those with chronic conditions but stratified by gender and age.

**Supplemental Table S2** Results of regression analysis: influence of age, gender, and presence of chronic health conditions on the QOLIBRI-OS-KID/ADO total score in the general population sample.

| **Variable (reference category in *italics*)** | **Estimate** | **S.E.** | ***t*** | ***p*** |
| --- | --- | --- | --- | --- |
| Intercept | 70.85 | 1.85 | 38.31 | **<0.001** |
| Age group  (Adolescents: 13–17 years \| *Children: 8–12 years*) | -7.64 | 2.2 | -3.48 | **0.001** |
| Gender  (Male \| *Female*) | 1.3 | 2.2 | 0.59 | 0.553 |
| Health status  (No chronic health conditions \| *At least one chronic health condition*) | 9.08 | 1.93 | 4.71 | **<0.001** |
| Age group * Gender | 3.73 | 1.46 | 2.55 | **0.011** |
| Age group * Health status | 5.2 | 2.21 | 2.35 | **0.019** |
| Gender * Health status | -2.84 | 2.21 | -1.28 | 0.199 |
| *Note*. *: interaction between the variables (for reference categories, see groups in *italics*); Estimate: non-standardized regression coefficient; S.E.: standard error; t: t-value; p: p-value; values in bold are significant at 5%. | | | | |

# References

1. von Steinbuechel N, Wilson L, Gibbons H, et al (2012) QOLIBRI Overall Scale: a brief index of health-related quality of life after traumatic brain injury. J Neurol Neurosurg Psychiatry 83:1041–1047. https://doi.org/10.1136/jnnp-2012-302361
